# Digital dermoscopy: a complementary method in the diagnosis of scabies^☆^^☆☆^

**DOI:** 10.1016/j.abd.2019.11.014

**Published:** 2020-06-26

**Authors:** Elaine Dias Melo, Carla Barros da Rocha Ribas, Isabel Cristina Lima Encarnação

**Affiliations:** a Teaching and Research Department, Fundação de Dermatologia Tropical e Venereologia Alfredo da Matta, Manaus, AM, Brazil; b Pediatric Dermatology Clinic, Fundação de Dermatologia Tropical e Venereologia Alfredo da Matta, Manaus, AM, Brazil

**Keywords:** Dermoscopy, Diagnosis by image, Scabies

## Abstract

Scabies is an ectoparasitosis caused by *Sarcoptes scabiei var. hominis*, characterized clinically by pruritic lesions in typical locations; the crusted form is a rare manifestation. The diagnosis is usually established based on the clinical picture, but dermoscopy can be an important complementary method, as it allows the observation of a brownish triangular structure with a hang-glider appearance. A case of crusted scabies is reported; the magnification of the images obtained by digital dermoscopy allowed the demonstration of a structure usually observed only with videodermoscopy.

Scabies is an ectoparasitosis caused by the mite *Sarcoptes scabiei var. hominis*, whose transmission occurs through interpersonal contact and occasionally through fomites.^1^, ^2^ Clinically, it is characterized by erythematous papules or vesicles and tunnels located in the interdigital spaces, flexor surfaces of the wrists, umbilical region, and flexural areas.^1^

The crusted form is considered a rare presentation; immunodepression and mental and/or motor deficits are the main risk factors.^3^ It manifests with hyperkeratotic, fissured, crusted, or erythrodermic lesions, variable pruritus, and the presence of millions of mites, being highly contagious.^1^, ^4^

The diagnosis is usually established based on the clinical picture, but it can be complemented by dermoscopy, videodermoscopy, or confocal microscopy, and is confirmed by direct examination or biopsy.^1^, ^2^ The authors report a case of crusted scabies with an emphasis on dermoscopic findings.

A female patient, 29 years of age, native Brazilian, presented with erythematous papules and lichenified plaques, some of which were hyperkeratotic, in the acral, flexural, and abdominal regions, associated with mild pruritus for eight months (Fig. 1). Dermoscopy was performed with immersion, under polarized light (10×, DermLite DL4, California, United States) of an unscathed lesion on the arm, and the images were captured with a camera (12 Mp, iPhone 7, Apple Inc., California, United States) with 2× optical zoom allowing the observation of the millipede-like structures and the hang-glider appearance (Fig. 2). Subsequently, image amplification demonstrated a translucent rounded structure (Fig. 3). Direct examination confirmed the diagnosis of crusted scabies (Fig. 4) and, after treatment, the patient evolved with resolution of the lesions.^5^

**Figure 1:**
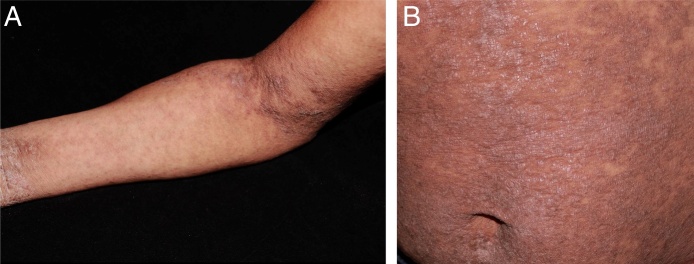
Erythematous papules and lichenified plaques, some hyperkeratotic, located mainly on the limbs, flexural areas (A), and abdomen (B).

**Figure 2:**
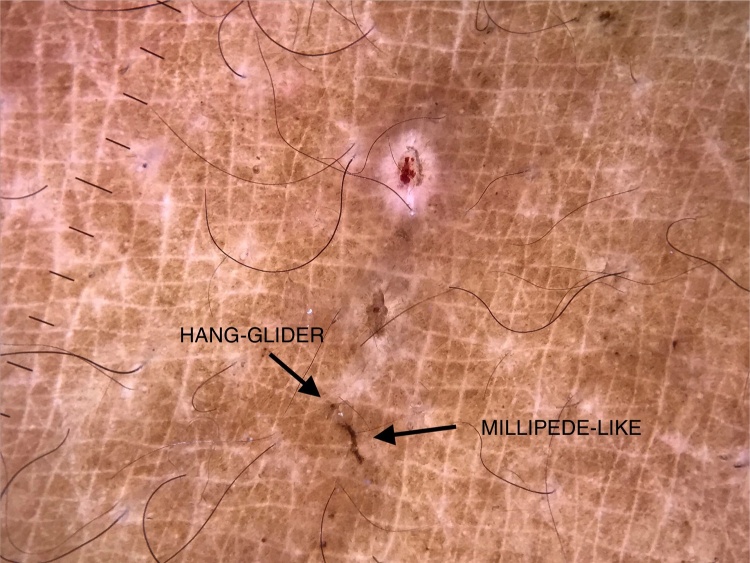
Demonstration of the structures: (i) hang-glider: anterior portion of the body and front legs of the mite; (ii) millipede-like: scabies tunnel; (iii) rounded translucent: mite body. (Digital dermoscopy, ×10 with immersion, under polarized light and optical zoom, ×2.)

**Figure 3:**
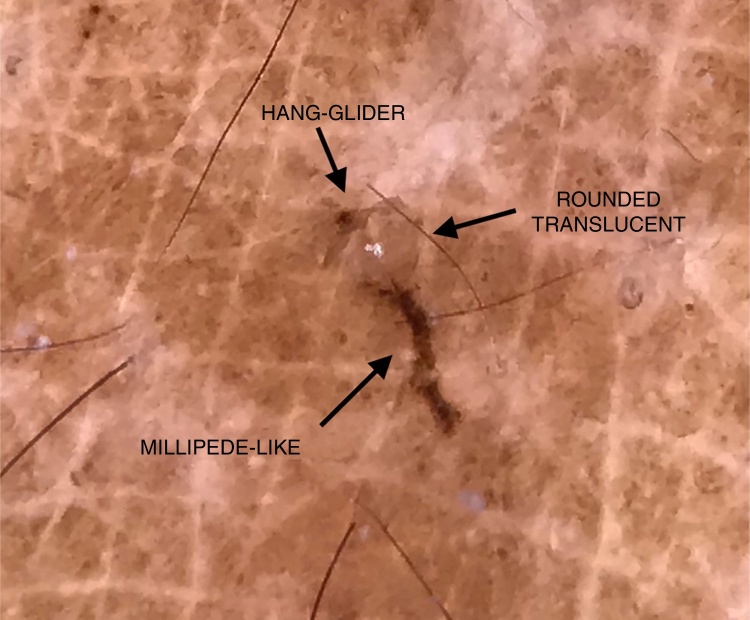
Demonstration of the structures: (i) hang-glider: anterior portion of the body and front legs of the mite; (ii) millipede-like: scabies tunnel; (iii) rounded translucent: mite body. (Digital dermoscopy, ×10 with immersion, under polarized light, optical zoom, ×2, and image amplification.)

**Figure 4:**
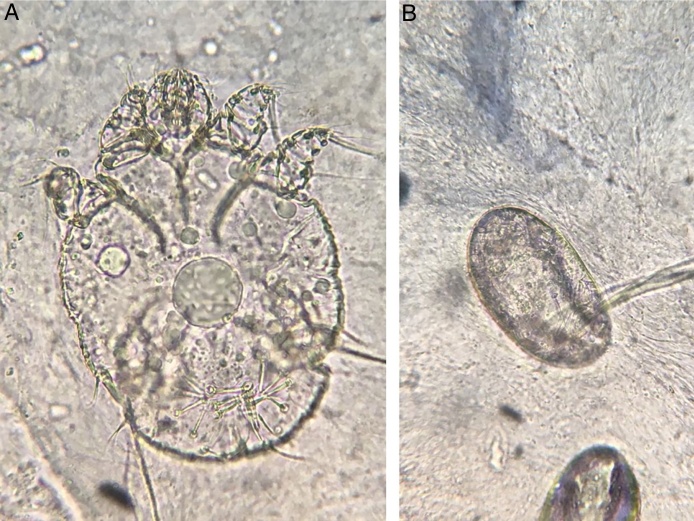
Observation of the mite (a) and eggs (b) in the direct examination (KOH 20%, ×100).

Dermoscopy has been widely reported in recent years as an aid in the diagnosis of scabies. It allows the observation of a brownish triangular structure with a hang-glider appearance, corresponding to the anterior portion of the body and the front legs of the mite, and a tunnel, which can assume a millipede-like conformation.^6^ In crusted scabies, the literature features reports of a tunnel-over-tunnel pattern, described as a noodle pattern, which corresponds to tunnels dug by various parasites.^7^

Observation of the mite's body is reported when using videodermoscopy.^2^, ^8^ In 2016, Cinotti et al. compared 20× and 70× magnifications, and only saw the body of the mite in the latter.^9^

However, in 2019, Scanni demonstrated this structure through digital dermoscopy, defined as acquisition and storage of images obtained by dermoscopy.^10^ That author associated dermoscopy (10×), optical zoom of the camera (3–5×), and image amplification, and demonstrated some structures that make up what he defined as “the mite-gallery unit.”^10^ This unit consists of the head, which corresponds to the mite with the hang-glider structure, the body, which contains the tunnel with eggs and feces, and the tail, the end of the tunnel composed of keratin collars, viewed only with dermoscopy without immersion. Through image amplification, the translucent body of the mite with several scattered dark spots (termed ladybird sign) can also be observed.^10^ In the present report, using this technique, it was possible to demonstrate the hang-glider appearance, the ovoid body, and the tunnel.

It is known that the observation of the mite body, eggs, and feces is the best with videodermoscopy.^2^, ^8^ However, when unavailable, the use of the technique described by Scanni and applied in the present report can allow a more accurate diagnosis of scabies.

## Financial support

None declared.

## Authors' contributions

Elaine Dias Melo: Conception and planning of the study; elaboration and writing of the manuscript; obtaining, analyzing, and interpreting the data; intellectual participation in propaedeutic and/or therapeutic conduct of studied cases; critical review of the literature.

Carla Barros da Rocha Ribas: Approval of the final version of the manuscript; elaboration and writing of the manuscript; effective participation in research orientation; intellectual participation in propaedeutic and/or therapeutic conduct of studied cases; critical review of the manuscript.

Isabel Cristina Lima Incarnation: Approval of the final version of the manuscript; effective participation in research orientation; intellectual participation in propaedeutic and/or therapeutic conduct of studied cases; critical review of the manuscript.

## Conflicts of interest

None declared.
